# A multi-view genomic data simulator

**DOI:** 10.1186/s12859-015-0577-1

**Published:** 2015-05-12

**Authors:** Michele Fratello, Angela Serra, Vittorio Fortino, Giancarlo Raiconi, Roberto Tagliaferri, Dario Greco

**Affiliations:** 1 Department of Medical, Surgical, Neurological, Metabolic and Ageing Sciences, Second University of Napoli, Napoli, Italy; 2 Department of Computer Science, Fisciano, Italy; 3 Unit of Systems Toxicology and Nanosafety Research Centre, Finnish Institute of Occupational Health, FIOH, Helsinki, Finland

**Keywords:** Multi-view, Regulatory network, Gene-miRNA interactions, OMICs data simulation

## Abstract

**Background:**

OMICs technologies allow to assay the state of a large number of different features (e.g., mRNA expression, miRNA expression, copy number variation, DNA methylation, etc.) from the same samples. The objective of these experiments is usually to find a reduced set of significant features, which can be used to differentiate the conditions assayed. In terms of development of novel feature selection computational methods, this task is challenging for the lack of fully annotated biological datasets to be used for benchmarking. A possible way to tackle this problem is generating appropriate synthetic datasets, whose composition and behaviour are fully controlled and known *a priori*.

**Results:**

Here we propose a novel method centred on the generation of networks of interactions among different biological molecules, especially involved in regulating gene expression. Synthetic datasets are obtained from ordinary differential equations based models with known parameters. Our results show that the generated datasets are well mimicking the behaviour of real data, for popular data analysis methods are able to selectively identify existing interactions.

**Conclusions:**

The proposed method can be used in conjunction to real biological datasets in the assessment of data mining techniques. The main strength of this method consists in the full control on the simulated data while retaining coherence with the real biological processes. The R package MVBioDataSim is freely available to the scientific community at http://neuronelab.unisa.it/?p=1722.

**Electronic supplementary material:**

The online version of this article (doi:10.1186/s12859-015-0577-1) contains supplementary material, which is available to authorized users.

## Background

OMICs technologies allow the comprehensive and parallel measurement of multiple molecular events (e.g., DNA modifications, RNA transcription and protein translation) in the same samples. Exploiting such complex and rich data is needed in the frame of systems biology for building global models able to explain complex phenotypes. In order to get useful information, the data must first be mined in search of relevant subsets of features, but classical feature selection methods can potentially fail as they classically test a feature at the time, not considering their potential interactions. Likewise, single-data layers (views) analysed separately could provide incomplete and fragmented information. On the contrary, multi-view leaning approaches take into account the different views simultaneously to reconstruct the underlying structure of the data. They can be benchmarked on real and synthetic datasets. A common problem with real datasets is that they are not fully understood and well annotated, whereas the synthetic data, although under full control, may be too simplistic to efficiently simulate the complex regulatory interactions among the molecules.

Different approaches for simulating biological data have been proposed. A first method consists in generating synthetic data with multivariate distributions similar to those observed on the real datasets [1-3]. New data can be generated using models that incorporate phenotypic variation, additive and multiplicative noise, transcriptional activity or inactivity, and/or block-correlation structures.

An alternative method focuses on generating data from synthetic transcriptional regulatory networks (TRNs). The main idea is to generate regulatory networks that include different types of biological interactions and produce biologically plausible synthetic gene expression data. An important point of these simulation methods is the computational technique used to quantitatively model the network interactions. A common technique for this purpose is based on solving a set of ordinary differential equations (ODEs) that explicitly model the variation of concentration of gene products. In [4-7], different models for the definition of the interactions are proposed.

In [5,6], interaction networks are sampled from existing ones. Starting from a given real network and a seed node of the network, a new network is constructed by sampling the modules of the real network. The main drawback of this method is that the number of possible networks that can be generated is limited by the size of the original network used for sampling. In [4], network topologies are generated based on different theoretical random network models. The main disadvantage of these models is that none of them can reproduce the characteristic of hierarchical modularity of TRNs. In [7], a hierarchical modular network is generated reproducing modules on different scales [8]. Starting with a network without connections, nodes are connected to each other following the patterns of known modules at different scales.

Once the topology is defined, interactions among the regulators are modelled by ODEs. In [4] interactions among regulators are modelled as the product of several Hill equations, one for each regulator. In [7] complex interactions among regulators like cooperation and competition are modelled with continuous Boolean logic functions.

None of these simulators is able to produce multi-view data, but provide a valuable source of techniques to be used for this purpose.

The state of a cell is regulated by a series of complex biological processes like protein synthesis, which is regulated by different control structures. The transcription factors (TF) are proteins that bind to specific regions of the genome regulating, together with other molecular signals such as histone modifications and DNA methylation, the transcription rate of the genes [9]. At the post-transcriptional level, microRNA (miRNA), whose transcription is also regulated similarly to the other genes, repress the protein expression [10].

*A priori* knowledge on the targeting patterns of TFs and miRNAs can be used, for instance, to produce network models of interaction. TRNs can be modelled as graphs in which nodes represent genes and edges represent the interactions between genes, such as activation or repression. Since the flow of information follows a precise direction, these graphs are directed. TRNs can be characterized by a set of global and local topological properties.

Similarly to other networks, also in TRNs, the degree distribution follows a power-law decay *P*(*k*)≈*k* ^−*α*^ with 2<*α*<3 [11,12]. This distribution is characteristic of the *scale-free* networks, in which the degree of a node is independent on the size (scale) of the network.

Another global characterization of TRNs is the clustering coefficient. For each node of the network it is defined as

(1) $$  C = \frac{n}{k \cdot \left(k - 1 \right)}  $$

where *n* is the number of connections between the neighbours and *k* is the number of neighbours. Studies have confirmed that the clustering coefficient in TRNs depends only on the degree of the nodes and it is distributed again as a power-law *C*(*k*)≈*k* ^−1^ [8,13,14]. Both these two properties specify that genes with low degree have a higher clustering coefficient than nodes highly connected leading to a hierarchical network of separated modules of genes interconnected by high-degree genes.

**Figure 1:**
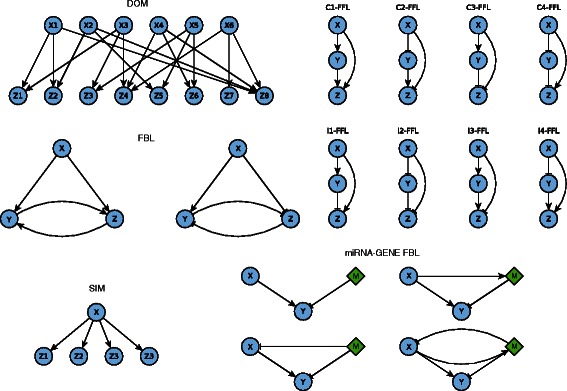
Motifs of interactions. Graphical representation of the interactions between genes and miRNAs. Arrows are for activation, blunt edges are for repression.

On a local scale genes organize in modules. The most significantly frequent patterns of connections between genes of a module are called *motifs* [15] each with different dynamical proprieties, such as self-regulation, feed-forward and feed-back loops and dense overlapping regulons [15,16] (Figure 1). The most frequent motifs that comprehend miRNAs and TFs interactions are the feedback and feed forward loops [17-19].

## Methods

Intuitively, network construction is based on an iterative procedure. The key idea is to construct a regulatory network starting from a graph without edges in which each node represent a gene or a miRNA and to add connections between nodes imitating some well known motif randomly chosen. Every time a motif is constructed into the network, all the participating nodes are removed from the graph. Regulating genes of the constructed motifs are kept in a separate set of nodes, namely, *H*. When the graph remains without nodes, a new graph is constructed with the nodes stored in *H* again with no edges. The procedure then restarts. This iterative method goes on until there are no nodes. The reinsertion of the regulating genes ensures the creation of a modular hierarchy of nodes.

The methods here proposed have been implemented as an R [20] package freely available from (Additional file 1) http://neuronelab.unisa.it/?p=1722.

### Network topology

The idea of creating a modular hierarchical network by replicating the same module at different scales was proposed in [8]. In [7] this replication procedure was extended by constructing a network using a set of motifs instead of a single one replicated at different scales. In this work, this idea is further extended with the addition of the interactions among TFs and miRNAs with the objective of synthesizing multi-view biological data. A set of motifs containing both TF-TF, miRNA-TF, and TF-miRNA interactions are defined based on [11,15,17], and recursively used as local templates to construct a network that satisfies the condition of hierarchical modularity.

The procedure starts with a network *N*=(*V*_*N*_,*E*_*N*_) of *n* genes and *m* miRNAs, with *n*+*m*=|*V*_*N*_|, and without edges *E*_*N*_=*∅*. In each step a pool of random motifs is generated. For each motif a score *S* is computed. This score measures the reduction in the difference between the degree distribution specified by the user and the current degree distribution. The score is the sum over a set of sub-scores

(2) $$  S\left(M\right) = \sum_{\substack{i \in \mathsf{genes}\left(V_{N}\right) \\ j \in \mathsf{genes}\left(V_{M}\right)}} Sg_{ij} + \sum_{\substack{i \in \mathsf{mirnas}\left(V_{N}\right) \\ j \in \mathsf{mirnas}\left(V_{M}\right)}} Sm_{ij}  $$

Each sub-score indicates the advantage of connecting node *i* in *V*_*N*_ as each node *j* in *V*_*M*_. For each *i* ∈ *genes*(*V*_*N*_) and *j* ∈ *genes*(*V*_*M*_), the sub-score is given by

(3) $$  Sg_{ij}=\sum\limits_{k=1}^{\vert V_{N} \vert} Sg_{ijk}  $$

Where *S* *g*_*ijk*_ is calculated by

(4) $$  Sg_{ijk}=\mathsf{sign}\left(\vert d^{\mathrm{p}}_{k} - p_{k} \vert - \vert d^{\mathrm{p}}_{k} - f_{kij} \vert \right) \cdot \frac{\vert d^{\mathrm{p}}_{k} - p_{k} \vert}{d^{\mathrm{p}}_{k}}  $$

in which $d^{\mathrm {p}}_{k}$ is the portion of nodes with degree *k* that is sampled from a power-law with parameter *α* specified as input by the user; *p*_*k*_ is the current portion of nodes with degree *k* and *f*_*kij*_ is the portion of nodes with degree *k* if node *i* gets the connections of node *j*.

The *sign*(·) factor determines whether adding the connections of node *j* to node *i* is a good decision (*sign*(·)>0) or not (*sign*(·)<0). The factor $\frac {\vert d^{\mathrm {p}}_{k} - p_{k} \vert }{d^{\mathrm {p}}_{k}}$ determines the magnitude of the advantage or disadvantage of edge additions to *N*, normalized by the number of desired nodes of degree *k*.

Sub-scores for nodes *i* ∈*mirnas*(*V*_*N*_) and *j* ∈*mirnas*(*V*_*M*_) are computed differently since miRNA-gene interactions respect different properties. The final portion of nodes regulated by a miRNA is denoted by $d^{\mathrm {e}}_{k}$, that is sampled from an exponential distribution of parameter *λ* given as input [18]. Whereas the desired number of nodes that regulate a miRNA is sampled by a power-law as in the previous case.

Globally considered,

(5) $$  Sm_{ij} = \sum\limits_{k=1}^{\vert \mathsf{mirnas}\left(V_{N}\right)\vert} Sm^{\text{in}}_{ijk} + Sm^{\text{out}}_{ijk}  $$

with

(6) $$  Sm^{\text{in}}_{ijk}=\mathsf{sign}\left(\vert d^{\mathrm{p}}_{k} - p^{\text{in}}_{k} \vert - \vert d^{\mathrm{p}}_{k} - f^{\text{in}}_{kij} \vert \right) \cdot \frac{\vert d^{\mathrm{p}}_{k} - p^{\text{in}}_{k} \vert}{d^{\mathrm{p}}_{k}}  $$

and

(7) $$  Sm^{\text{out}}_{ijk}=\mathsf{sign}\left(\vert d^{\mathrm{e}}_{k} - p^{\text{out}}_{k} \vert - \vert d^{\mathrm{e}}_{k} - f^{\text{out}}_{kij} \vert \right) \cdot \frac{\vert d^{\mathrm{e}}_{k} - p^{\text{out}}_{k} \vert}{d^{\mathrm{e}}_{k}}  $$

Given the score for each motif in the pool a motif is selected by sampling a distribution proportional to the scores. The selected motif is used as a template. A subset of nodes of the current network *N* are sampled using the sub-scores *S* *g*_*ij*_ and *S* *m*_*ij*_ and are connected as the nodes in the motif.

During each edge addition, a set of parameters is generated in order to characterize the dynamical properties of the interaction and making the overall behaviour of the motif similar to its real-world counterparts. For example, using the same terminology of [15], the Single-input motif (Figure 1), is considered to generate coordinated expression of a set of genes, and, more interestingly scheduled expression schemas, in which the regulated genes will express in a defined order.

The selected nodes are then removed from *N* and in a separate set *H*, which is initially empty, are added the nodes that took the role of x in Figure 1. When there are no more nodes to connect in *V*_*N*_, the nodes in *H* are passed into *V*_*N*_, *H* is set to *∅* and a new iteration is started. This process goes on until both *V*_*N*_ and *H* are empty.

Each time *V*_*N*_ gets the nodes of *H*, modules of nodes in the network get connected hierarchically.

When the network construction is completed, a special class of nodes are added to the network: signalling nodes. These nodes are responsible of transferring information to the network [15,21]. Stimulation signals are an example of information passed. System state can be set through signals as covered in the next section. The number of signalling nodes to be placed in the network is determined by the user. Signalling nodes only have outgoing edges. Target genes are determined sampling a distribution proportional to the out degree of the nodes of the network. This ensures that the majority of genes controlled by signals, have enough capability of controlling the state of the network during simulation.

A more concise representation of the network generation procedure is reported in Algorithm 1.



### Simulation

Simulation of the system is based on ODEs. Concentrations of gene products are modelled by continuous variables on a limited time interval [22]. The rate of production of a given element *x*_*i*_ depends on the concentration of its regulatory components, both genes and miRNAs (to not clutter the notation we omit explicit time dependency of concentrations and concentration rates)

(8) $$  \frac{\mathrm{d}x_{i}}{\mathrm{d}t} = f_{i}(\mathbf{x}, \mathbf{m})  $$

**x** is the vector of concentrations of the genes regulating *x*_*i*_, **m** is the vector of concentration levels of the miRNAs regulating *x*_*i*_ and *f*_*i*_ is a non-linear regulation function of these components. A common model for *f*_*i*_(**x**,**m**) with a single regulating gene *x*_*j*_ and a single miRNA *m*_*k*_ is

(9) $$  \frac{\mathrm{d}x_{i}}{\mathrm{d}t} = p_{i} \cdot r_{i} \left(x_{j}\right) - d_{i}\left(m_{k} \right) \cdot x_{i}  $$

Where *p*_*i*_ is the basal production rate of *x*_*i*_, i.e., the basic rate of production; *r*_*i*_(*x*_*j*_) is the function that model the regulation of *x*_*j*_ on *x*_*i*_ and *d*_*i*_(*m*_*k*_) is the degradation function [22,23] that depends on the concentration level of *m*_*k*_.

**Figure 2:**
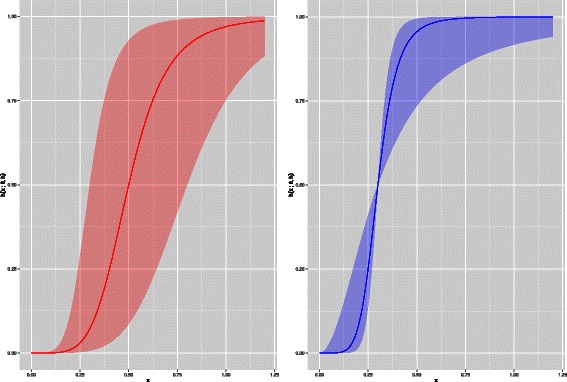
Hill functions. Shapes of the Hill function for different values of the parameters. The solid red line is a Hill function with parameters *θ*=0.5 and *μ*=5. The shaded red area is the family of Hill functions obtained when *θ*∈[0.3,0.8] and *μ* is fixed. Similarly, the solid blue line is the Hill function of parameters *θ*=0.3 and *μ*=6. The shaded blue area is the family of Hill functions obtained when *μ*∈[2,10] and *θ* is fixed.

A common regulation function is the *Hill equation* [24]

(10) $$  h\left(x_{j}; \theta, \mu\right) = \frac{x_{j}^{\mu}}{x_{j}^{\mu} + \theta^{\mu}}  $$

with *h*(*x*_*j*_;*θ*_,_ *μ*)∈[0,1]. Parameter *θ*>0 is the value at which *h*(*x*_*j*_;*θ*_,_ *μ*)=0.5, i.e., a threshold on the concentration level of *x*_*j*_; *μ*>0 controls the steepness of the function. For *μ*>1 the Hill equation has a sigmoid shape (Figure 2).

The degradation rate of target genes is directly influenced by the regulating miRNA *m*_*k*_ [25,26]. The degradation function is defined as

(11) $$  d_{i}\left(m_{k} \right) = d_{i0} + d_{i} \cdot h\left(m_{k}; \theta, \mu\right)  $$

The first term is the basal degradation rate, that is the rate of degradation of *x*_*i*_ independent of *m*_*k*_ and *d*_*i*_ is the rate of degradation dependent on the concentration of *m*_*k*_.

The miRNA rate of production is assumed to follow a law similar to the production of genes, but with a constant degradation rate.

When there’s more than a regulator the Hill equation will not suffice. Hence, there is the need for a model taking into account interactions among regulators in addition to interactions between regulators and the regulated gene. Since most of the interactions among regulators are unknown [15], we apply the same idea proposed in [7] and define the possible interactions among regulators by combinations of simple functions. Here we follow the same approach and define the same simple interaction functions among regulators:

- **Cooperation**
  All regulators need to be highly expressed to activate the regulated gene
  (12) $$\begin{array}{@{}rcl@{}}  &\mathsf{COOP}\left(x_{1}, \ldots, x_{n}\right)=\mathsf{min}\left(h\left(x_{1}\right), \ldots, h\left(x_{n}\right) \right)& \end{array} $$
- **Synergy**
  Contemporary activation of all regulators is not necessary to activate the regulated gene
  (13) $$\begin{array}{@{}rcl@{}} &\mathsf{SYN}\left(x_{1}, \ldots, x_{n}\right)=\mathsf{min}\left(1, h\left(x_{1}\right) + \ldots + h\left(x_{n}\right) \right)& \end{array} $$
- **Inhibition**
  Activation of the regulator means target gene is repressed
  (14) $$\begin{array}{@{}rcl@{}} &\mathsf{INH}\left(x\right)=1-h\left(x\right)& \end{array} $$
- **Competition**
  Regulator *x*_1_ competes with repressor *x*_2_
  (15) $$\begin{array}{@{}rcl@{}} &\mathsf{COMP}\left(x_{1}, x_{2}\right)=\mathsf{max}\left(0, h\left(x_{1}\right) - h\left(x_{2}\right) \right)& \end{array} $$

It is to be noted that in this case the threshold and steepness parameters are different for each interaction. The specific regulation function of each gene is defined by the composition of randomly sampled functions and the regulators that will interact.

Since miRNAs tend to increment the rate of degradation of target genes, resulting in reduced expression levels, we assume that the only type of interaction among miRNAs regulating the same target gene is a synergyc inhibition.

Once all the system parameters are specified, the set of ODEs is solved with a numerical procedure over a given time interval. An initial value for the system must be specified. The result of the simulation can be used both as a time series dataset, or as steady state microarray data by sampling the time series.

Different experimental conditions can be simulated using controlling signals for the synthetic subjects. A large set of different stimuli can be simulated, from inhibition of some hub gene (with a constant 0 signal) to periodic drug administration (using periodic signals).

### Variability of the model

In order to generate plausible expression values for different simulated subjects it must be present a degree of variability in the model. We used a two-level model comprehending biological and technical variability.

Biological variability is an intrinsic characteristic among beings of the same species and is implemented in the synthetic system as a small amount of noise in system parameters values. Specifically, white noise with low standard deviation is added for each subject to be simulated.

Technical variability is an inevitable part of the data acquisition process and is simulated implementing the model of error measurement proposed in [27] that considers two error components. For each true expression level *x*_*i*_, the measured intensity *y*_*i*_ is given by

(16) $$  y_{i} = c + x_{i}e^{\eta} + \epsilon  $$

where *c* is the constant mean background level. *ε* is an additive error term distributed as $\mathcal {N}\left (0, \sigma _{\epsilon } \right)$ that represents the background noise and mostly influences low expressed genes. The second error term is $\eta ~\sim ~\mathcal {N}\left (0, \sigma _{\eta }\right)$, a multiplicative factor that represents the proportional error that mostly influences higher expression values.

## Results and discussions

### Network validation

**Figure 3:**
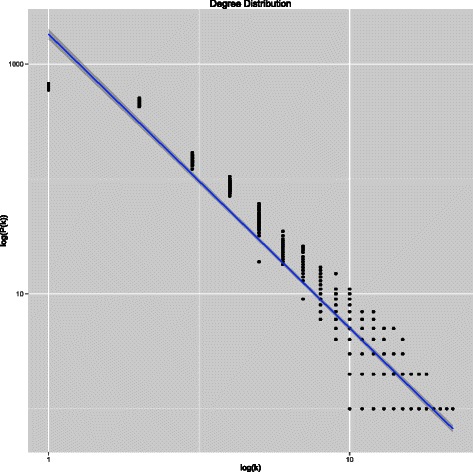
Fitting of degree distribution. The degree distribution of 50 networks generated with the same size is fitted by a line in log-log space. The resulting estimated scale parameter is $\hat {\alpha }=2.5566$ with *R* ^2^=0.9362.

In order to verify the hierarchical modularity of the generated regulatory networks, we constructed different sets of networks of different sizes with default parameters. The scale-free property has been verified generating 50 networks of 1000 nodes with the same scale parameter *α*=2.2. We measured the degree distribution for each network and fitted a line in the log-log plot. In Figure 3 is shown the resulting fit. From the generated networks we estimated a scale parameter $\hat {\alpha }=2.5566$ and a goodness-of-fit parameter of *R* ^2^=0.9362.

**Figure 4:**
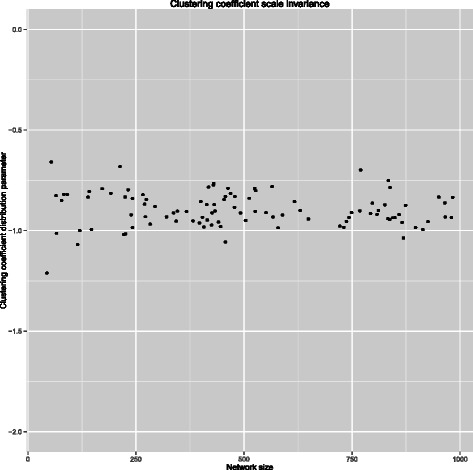
Scale invariance of clustering coefficient. Simulation of 100 networks of random size in [10,1000] shows that the estimated scaling parameter of the clustering coefficient is independent from the network size and approximates the value found in real networks.

We then verified the scale-invariance of the clustering coefficient. For this, we generated 100 networks with size randomly sampled from the interval [10,1000]. In Figure 4 the estimated scale parameter of the distribution of the clustering coefficient in relation with network size is shown. Together these results show that generated networks have the hierarchical modularity property of the real regulatory networks.

In the rest of this section we report four cases of analysis that can be performed on the generated datasets: two examples try to explore the topology of the network and are based on network reconstruction methods and on clustering methods. The other two are methods of feature relevance: a filter method based on *t*-tests and a wrapper method based on the Boruta method.

For the experiments we generated three regulatory networks from which we generated different simulated dataset of increasing complexity: **GRN1** 1000 genes, 100 miRNAs and 10 controlling signals **GRN2** 1000 genes, 300 miRNAs and 35 controlling signals **GRN3** 500 genes, 100 miRNAs and 20 controlling signals

In all cases the synthetic datasets are obtained by simulating the regulatory network for an amount of 100 time points. The resulting dataset is obtained by taking the expression values at the last simulated time point.

### Reverse engineering

We wanted to test if the synthetic networks generated with the proposed model can be reconstructed with commonly used tools for this task. From **GRN1** we generated a dataset of 75 samples by assigning to each of the 10 controlling signals a constant value randomly sampled from a uniform distribution in [0,1]. We estimated the significance of each connection for both gene-only expression dataset and genes+miRNAs dataset with PANDA [28]. PANDA is a message-passing network prediction method based on interactions among TFs and regulated genes. Information for each type of interaction is propagated to the others iteratively, resulting in a prediction score for each interaction.

For both types of regulatory networks we provided different numbers of *a priori* connections. We executed PANDA with prior information covering the 10*%*, 25*%*, 50*%*, 75*%* and 100*%* of all actual connections among the gene-gene network (1656 edges) and full network interactions (3969 edges).

In addition, we introduced noisy prior information in the form of false connections. Different quantities of incorrect edges have been tested, namely 10*%*, 25*%*, 50*%*, 75*%*, 100*%* of incorrect edges.

Since the PANDA scores can be interpreted as *z*-scores, we set a *p*-value threshold to 0.05 for both nominal and Bonferroni corrected *p*-values. We also set a threshold of 0.05 to false discovery rate (FDR). For each significant connection we calculated the length of the path in the actual synthetic network.

In Tables 1, 2, 3 and 4 are reported the results of the analysis (where 1 signifies direct interaction, >1 signifies indirect interaction, Inf signifies no interaction). When given correct prior information PANDA is able to mark as significant almost 100*%* of true interactions, whereas when noisy (false) prior information is passed, none of it is marked as significant.

**Table 1:** **Gene-only path length**

**Prior connections**	**p** **<0.05 (uncorrected) Path length**						**p** **<0.05 (Bonf.) Path length**	**FDR** **<0.05 Path length**
	**0**	**1**	**2**	**3**	**5**	**Inf**	**1**	**1**
165 true	-	166	1	-	-	1	165	165
414 true	-	414	-	-	-	2	414	414
828 true	-	829	7	1	-	4	828	828
1242 true	1	1243	3	-	1	5	1242	1242
1656 true	-	1656	2	-	-	3	1656	1656

Path length of significant interactions confirmed by PANDA on the gene-only regulatory network with different amounts of correct prior information.

**Table 2:** **Gene-only path length with false information**

**Prior connections**	**p** **<0.05 (uncorrected) Path length**				**p** **<0.05 (Bonf.) Path length**	**FDR** **<0.05 Path length**
	**1**	**2**	**5**	**Inf**	**1**	**1**
1656 true + 165 false	1656	1	-	8	1656	1656
1656 true + 414 false	1656	2	1	7	1656	1656
1656 true + 828 false	1656	-	-	4	1654	1656
1656 true + 1242 false	1656	2	1	7	1653	1656
1656 true + 1656 false	1656	2	-	4	1647	1656

**Table 3:** **Whole-network path length**

**Prior connections**	**p** **<0.05 (uncorrected) Path length**							**p** **<0.05 (Bonf.) Path length**	**FDR** **<0.05 Path length**
	**0**	**1**	**2**	**3**	**4**	**5**	**6**	**1**	**1**
396 true	-	396	-	-	-	-	-	396	396
992 true	1	993	-	-	1	1	-	992	992
1984 true	1	1985	-	2	2	1	1	1984	1984
2976 true	-	2976	3	-	3	2	1	2976	2976
3969 true	-	3969	-	1	1	-	-	3969	3969

Path length of significant interactions confirmed by PANDA on the whole regulatory network with different amounts of correct prior information.

**Table 4:** **Whole-network path length with false information**

**Prior connections**	**p** **<0.05 (uncorrected) Path length**				**p** **<0.05 (Bonf.) Path length**	**FDR** **<0.05 Path length**
	**1**	**2**	**3**	**4**	**1**	**1**
3969 true + 396 false	3969	-	-	1	3671	3930
3969true + 992 false	3969	-	-	1	3653	3918
3969 true + 1984 false	3969	-	1	-	3640	3899
3969 true + 2976 false	3969	-	1	1	3655	3874
3969 true + 3969 false	3968	1	1	1	3659	3859

Path length of significant interactions confirmed by PANDA on the whole regulatory network with the presence of different amounts of noisy prior information.

We carried out additional tests using ARACNE [29], which estimates pairwise interactions by the degree of mutual information shared among the nodes in exam. Indirect connections that may stem are removed applying the data processing inequality. Starting from the expression dataset we estimated the mutual information matrix for both gene-only interactions and for the full regulatory network. We set a threshold of 0.05 on the weights of the reconstructed connections and checked how many of them are actual connections in the synthetic network.

In Table 5 are listed the path lengths for the interactions predicted by ARACNE on the synthetic network. In the gene-only network, most of the interactions found do not actually exist, whereas in the full network, comprising both genes and miRNAs, about half of the interactions found exist in the network but have an average path length of 4.38.

**Table 5:** **Whole-network path length with ARACNE**

	**Path length**									
	**1**	**2**	**3**	**4**	**5**	**6**	**7**	**8**	**9**	**Inf**
Gene-only Interactions	25	8	9	6	4	3	-	-	-	6493
Whole-network Interactions	43	150	681	1342	1367	449	130	12	2	3704

Path length of interactions inferred by ARACNE on the gene-only and full regulatory networks.

The high rate of erroneous interactions may be due to the fact that ARACNE works well when the role of the loops in the regulatory network is negligible [29], whereas the networks produced by the proposed simulator involve both feedback and feed-forward loops on different scales (i.e., from loops of nodes to loops of motifs) that may produce complex behaviours like oscillations or memory states. In addition, miRNAs also participate in loops with genes. Both facts may motivate the high levels of false interactions found in the gene-only network, where the miRNA layer of information needed to explain the behaviour of genes is not included in the analysis.

We speculate that the large number of direct interactions inferred by ARACNE in the full regulatory network may be due to the simplistic model of variation employed. This results in nodes of the same pathway sharing too much information, such that they look like directly connected with respect to the Mutual Information.

### Clustering of genes and miRNAs

Broadly speaking, clustering a set of objects aims to partition them into disjoint subsets. This partition is such that objects from different subsets are as much dissimilar as possible, whereas objects of the same cluster are maximally similar. Clustering has been widely applied to gene expression profiles across subjects. Gene clustering can be used as a mean of dimensionality reduction technique in which only a representer for each cluster is used instead of the entire dataset for further analysis [30]. In addition, gene clustering can be useful to predict the functional role of unknown genes based on the known genes of the same cluster [31].

We analysed two different synthetic datasets. The first dataset was generated from **GRN2**. The dataset is made of two classes each of 50 samples. The signalling genes were all set to 0 for the first condition and to 1 for the second condition (relative expression levels).

The second dataset is made of 75 samples divided into three classes of 25 samples each. The dataset is simulated from **GRN1**. For each condition we defined a constant expression value for the 10 controlling genes by randomly sampling a uniform distribution $\mathcal {U}\left (0,1\right)$. In both experiments for each sample we add a small amount of white noise to network parameters and then we simulated the network over an interval of 100 time points and taking as the expression dataset the last time-point.

**Figure 5:**
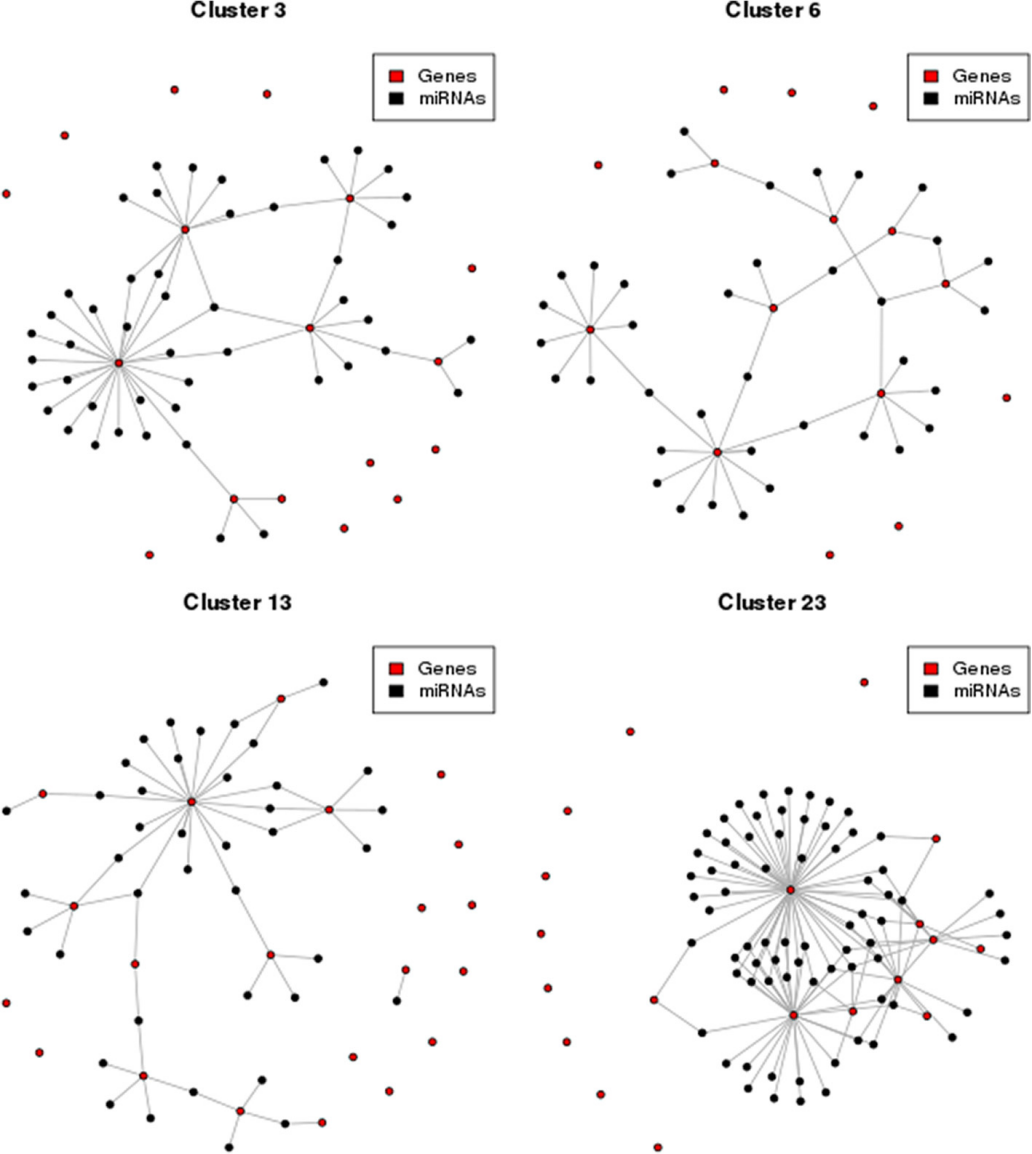
Revealed interactions among clustered genes. Clustered genes and miRNAs together with interactions. The majority of nodes that are clustered together are actually connected in the network from which data has been simulated.

For both synthetic datasets we used the *k*-means clustering algorithm on the features (genes and mirnas). Genes and miRNAs of both datasets have been standardized so that the mean of each gene and miRNA is 0 and the standard deviation is 1, then clustered into 50 groups. Data standardization makes the differences among genes and miRNAs depend on their correlations. In Figure 5 are shown some of the clustered genes and miRNAs along with the information coming from the known regulatory network (the actual connections). As can be seen nodes (genes and miRNAs) that are clustered together are actually connected in the network from which the data has been generated.

### Feature relevance

Due to the high-dimensional nature of OMICs data, effective modelling for inference or prediction in bioinformatics cannot be performed without an initial phase of feature selection. Different approaches to feature selection are available, which can be summarized in three categories: filter, wrapper and embedded methods, each with its own advantages and disadvantages [30]. We performed two feature relevance analysis. The first dataset is made of 50 samples for each condition (2 conditions in total) generated from **GRN3**. In this experiment we wanted to simulate the case in which the 2 different conditions are well characterized by a subset of controlling signals in the form of an expression signature by setting 13 of the 20 control signals to a deterministic value. Specifically, the first 13 genes for the first condition have been set to values 1100011011011 mixed with the remaining 7 control signals set to random values sampled from $\mathcal {U}\left (0,1\right)$. For the second condition the first 13 genes have been set to 1001110100010. We applied a filter feature selection method based on the *t*-test [32]. We set a threshold on the FDR to 0.05 and marked as significant all the genes with a *q*-value below the threshold. 8 out of the 39 significant genes are actual signalling genes, 22 significant genes are at path length 1 from a signalling gene, the remaining 9 are at path length 2 from a signalling gene. The same has been made for miRNAs and 3 out of 5 are at distance 1 from a signalling gene and the remaining 2 are at distance 2.

The second dataset consists of 75 samples divided into three classes of 25 samples each generated from **GRN1** by setting the controlling signals to random constant values sampled from $\mathcal {U}\left (0,1\right)$. For this more complex dataset we used the wrapper method of Boruta [33]. This method relies on the random forest classifier. The significance of each feature is assessed comparing its importance given by the random forest to the importance of a randomly computed version of the same feature. Features that are significantly more important than their random permutations are marked as relevant.

The procedure marked as relevant 59 genes: 9 are signalling genes, 28 are directly connected to (at least) a signalling gene, 20 are at distance 2 from a signalling gene and the remaining 3 genes are at distance 3 from a signalling gene. Of the 6 relevant miRNAs, 5 are at distance 2 from a signalling gene and only 1 is at distance 1 from a signalling gene.

From these experiments it is to be noted that almost all signalling genes which have been set to different values for each experimental condition are recognised as significant. The remaining signalling genes that are not marked as significant may have been set to values too similar between different conditions or the amount of noise is such to deteriorate the pattern. It should be also noted that both feature relevance procedures marked as significant nodes directly connected to at least a signalling gene or in the same pathway. This shows the capability of the proposed model of propagating information through modules of locally connected genes.

## Conclusions

Here we proposed a multi-view biological data simulator based on ordinary differential equations with the objective of benchmarking multi-view learning methods. We ensured that the generated data is biologically relevant for the features need to follow patterns of interaction that are similar to those observed in real biological networks. We showed different cases of analysis where the simulated datasets can complement real datasets in the assessment of novel methods for data analysis. At the same time the sample analysis further validated the proposed approach since information coherent with the regulatory network is extracted from the synthetic dataset. It will be possible to implement additional layers of complexity (e.g., including DNA methylation or copy number variations) as more comprehensive and systematic knowledge on the biological interactions arises.

## Additional file

Additional file 1
**MVBioDataSim R package.** The R implementation of the proposed method is available as R package attached to this paper.

## Abbreviations

TRN: Transcriptional regulatory network
ODE: Ordinary differential equations
TF: Transcription factor
miRNA: microRNA
FDR: False discovery rate

## References

[1] Bian S, Wang W (2005). Computational intelligence and security. Lecture Notes in Computer Science. vol. 3801.

[2] Zhang J, Coombes K. UMPIRE: Ultimate Microarray Prediction, Inference, and Reality Engine. In: BIOTECHNO 2011, The Third International Conference on Bioinformatics, Biocomputational Systems and Biotechnologies: 2011. p. 121–125.

[3] Muselli M, Bertoni A, Frasca M, Beghini A, Ruffino F, Valentini G. A mathematical model for the validation of gene selection methods. IEEE/ACM Trans Comput Biol Bioinform; 8(5):1385–92. doi:10.1109/TCBB.2010.83. 10.1109/TCBB.2010.83 21778526

[4] Mendes P, Sha W, Ye K (2003). Artificial gene networks for objective comparison of analysis algorithms. Bioinformatics.

[5] Van den Bulcke T, Van Leemput K, Naudts B, van Remortel P, Ma H, Verschoren A, et al. SynTReN: a generator of synthetic gene expression data for design and analysis of structure learning algorithms. BMC Bioinformatics. 2006; 7:43. doi:10.1186/1471-2105-7-43. 10.1186/1471-2105-7-43 PMC1373604 16438721

[6] Schaffter T, Marbach D, Floreano D (2011). GeneNetWeaver: in silico benchmark generation and performance profiling of network inference methods. Bioinformatics (Oxford, England).

[7] Di Camillo B, Toffolo G, Cobelli C (2009). A gene network simulator to assess reverse engineering algorithms,. Ann N Y Acad Sci.

[8] Ravasz E, Somera aL, Mongru Da, Oltvai ZN, Barabási aL (2002). Hierarchical organization of modularity in metabolic networks. Science (N Y).

[9] Bernstein BE, Birney E, Dunham I, Green ED, Gunter C, Snyder M (2012). An integrated encyclopedia of DNA elements in the human genome. Nature.

[10] Ambros V (2004). The functions of animal microRNAs. Nature.

[11] Milo R, Shen-Orr S, Itzkovitz S, Kashtan N, Chklovskii D, Alon U (2002). Network motifs: simple building blocks of complex networks. Science (N Y).

[12] Thieffry D, Huerta AM, Pérez-Rueda E, Collado-Vides J. From specific gene regulation to genomic networks: a global analysis of transcriptional regulation in Escherichia coli. Bioessays; 20(5):433–0. doi:10.1002/(SICI)1521-1878(199805)20:5<433::AID-BIES10>3.0.CO;2-2. 10.1002/(SICI)1521-1878(199805)20:5<433::AID-BIES10>3.0.CO;2-2 9670816

[13] Barabási AL, Oltvai ZN (2004). Network biology: understanding the cell’s functional organization. Nat Rev Genet.

[14] Potapov AP, Voss N, Sasse N, Wingender E (2005). Topology of mammalian transcription networks. Genome Inform.

[15] Alon U (2007). Network motifs: theory and experimental approaches,. Nat Rev Genet.

[16] Shen-Orr SS, Milo R, Mangan S, Alon U (2002). Network motifs in the transcriptional regulation network of Escherichia coli. Nat Genet.

[17] Shalgi R, Lieber D, Oren M, Pilpel Y. Global and local architecture of the mammalian microRNA-transcription factor regulatory network. PLoS Comput Biol. 2007; 3(7):131. doi:10.1371/journal.pcbi.0030131. 10.1371/journal.pcbi.0030131 PMC1914371 17630826

[18] Martinez NJ, Ow MC, Barrasa MI, Hammell M, Sequerra R, Doucette-Stamm L (2008). A C. elegans genome-scale microRNA network contains composite feedback motifs with high flux capacity. Genes Dev.

[19] Sun J, Gong X, Purow B, Zhao Z. Uncovering MicroRNA and Transcription Factor Mediated Regulatory Networks in Glioblastoma. PLoS Comput Biol. 2012; 8(7):1002488. doi:10.1371/journal.pcbi.1002488. 10.1371/journal.pcbi.1002488 PMC3400583 22829753

[20] R Core Team. R: A Language and Environment for Statistical Computing. Vienna, Austria; 2014. http://www.r-project.org/.

[21] Hecker M, Lambeck S, Töepfer S, van Someren E, Guthke R (2009). Gene regulatory network inference: data integration in dynamic models-a review. Biosystems.

[22] de Jong H (2002). Modeling and simulation of genetic regulatory systems: a literature review. J Comput Biol.

[23] Karlebach G, Shamir R (2008). Modelling and analysis of gene regulatory networks. Nat Rev Mol Cell Biol.

[24] Hill A (1910). The possible effects of the aggregation of the molecules of haemoglobin on its dissociation curves. J Physiol (Lond).

[25] Vohradsky J, Panek J, Vomastek T (2010). Numerical modelling of microRNA-mediated mRNA decay identifies novel mechanism of microRNA controlled mRNA downregulation. Nucleic Acids Res.

[26] Khanin R, Vinciotti V (2008). Computational modeling of post-transcriptional gene regulation by microRNAs. J Comput Biol.

[27] Rocke DM, Durbin B (2001). A model for measurement error for gene expression arrays. J Comput Biol.

[28] Glass K, Huttenhower C, Quackenbush J, Yuan GC. Passing messages between biological networks to refine predicted interactions. PloS One. 2013; 8(5):64832. doi:10.1371/journal.pone.0064832. 10.1371/journal.pone.0064832 PMC3669401 23741402

[29] Margolin AA, Nemenman I, Basso K, Wiggins C, Stolovitzky G, Dalla Favera R, et al. ARACNE: an algorithm for the reconstruction of gene regulatory networks in a mammalian cellular context. BMC Bioinformatics. 2006; 7((Suppl 1)):7. doi:10.1186/1471-2105-7-S1-S7. 10.1186/1471-2105-7-S1-S7 PMC1810318 16723010

[30] Guyon I, Elisseeff A (2003). An introduction to variable and feature selection. J Machine Learn Res.

[31] D’haeseleer P (2005). How does gene expression clustering work?. Nat Biotechnol.

[32] Saeys Y, Inza In, Larrañaga P (2007). A review of feature selection techniques in bioinformatics. Bioinformatics.

[33] Kursa MB, Jankowski A, Rudnicki WR (2010). Boruta - A system for feature selection. Fundamenta Informaticae.

