# Supplementary material for: A multi-view genomic data simulator
Source: BMC Bioinformatics. 2015 May 12;16:151. doi: 10.1186/s12859-015-0577-1 (PMC4448275; doi:10.1186/s12859-015-0577-1)
Supplement: Additional file 1 — MVBioDataSim R package. The R implementation of the proposed method is available as R package attached to this paper. [file 12859_2015_577_MOESM1_ESM.zip › MVBioDataSim_0.1.tar.gz!/MVBioDataSim/examples/hill.pdf]

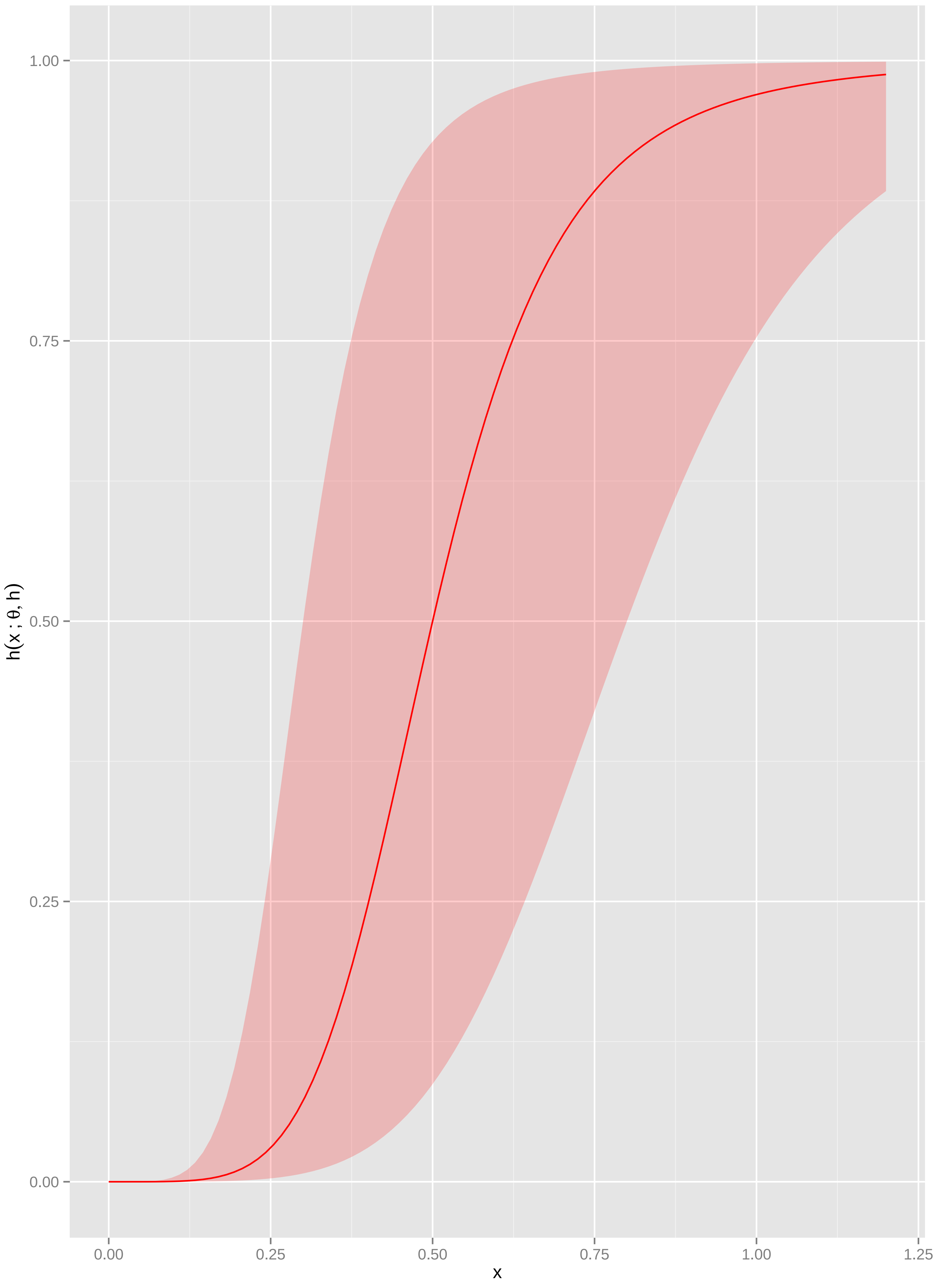

h(x ; θ, h)
1.00
0.75
0.50
0.25
0.00
0.00
0.25
0.50
0.75
1.00
1.25
x

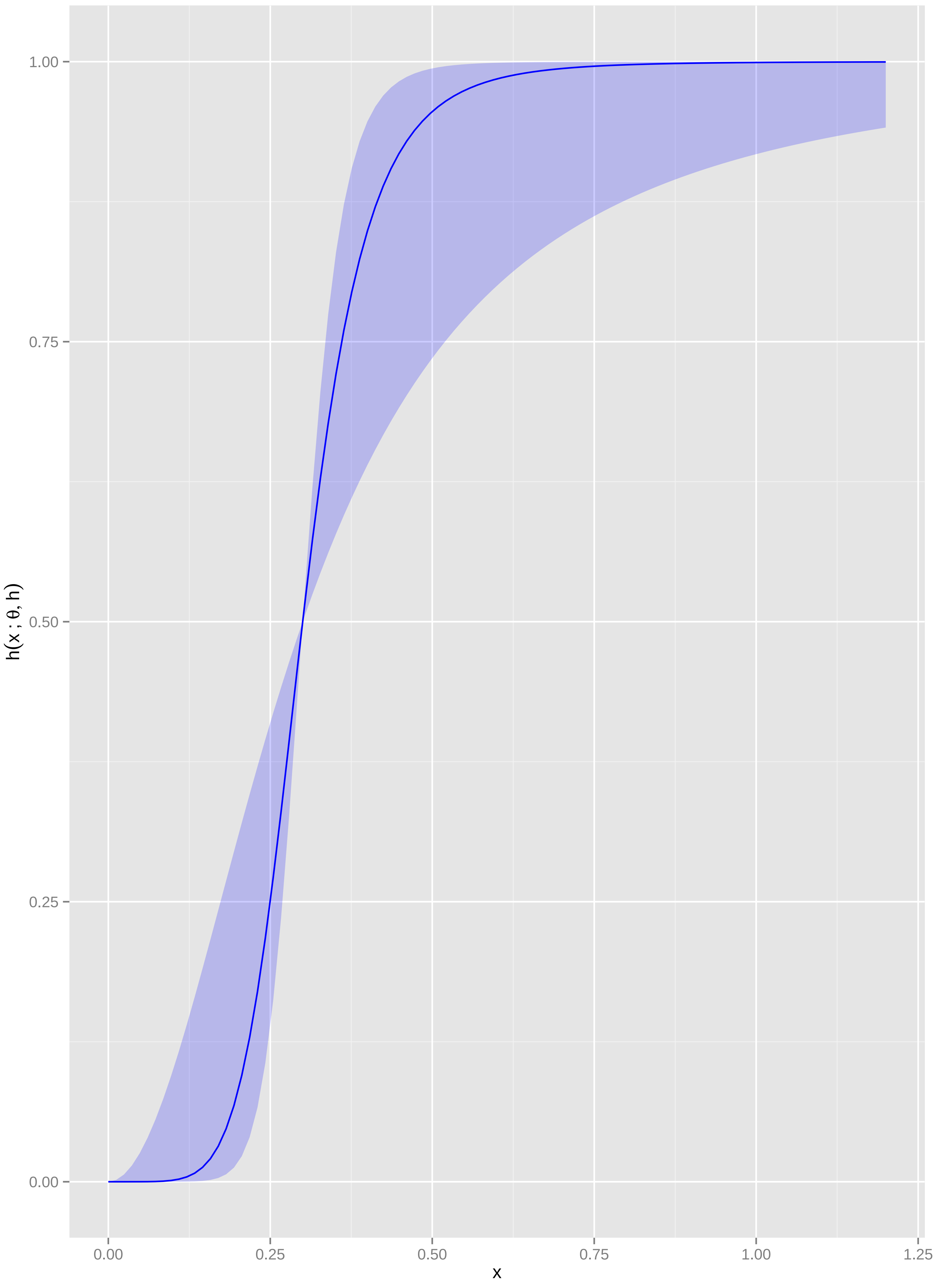

h(x ; θ, h)
1.00
0.75
0.50
0.25
0.00
0.00
0.25
0.50
0.75
1.00
1.25
x
